# The Yellow Colouring Matter of the Bile

**Published:** 1846-03

**Authors:** 


*The Yellow Colouring Matter of the Bile.*—M. Polli read a paper at the Congress of Italian Savans at Naples last year, detailing certain experiments made by him on the yellow colouring matter of the bile, and on the colouring matter of the blood, from which he draws the conclusion that these two principles are identical in their nature, but differ in their degree of oxidation, that of the blood being at the maximum, and that of the bile at the minimum.—*Ibid.*

